# Divergent Roles of Vascular Burden and Neurodegeneration in the Cognitive Decline of Geriatric Depression Patients and Mild Cognitive Impairment Patients

**DOI:** 10.3389/fnagi.2017.00288

**Published:** 2017-09-01

**Authors:** Qing Ye, Fan Su, Liang Gong, Hao Shu, Wenxiang Liao, Chunming Xie, Hong Zhou, Zhijun Zhang, Feng Bai

**Affiliations:** Department of Neurology, Affiliated ZhongDa Hospital, School of Medicine, Southeast University Nanjing, China

**Keywords:** cognitive decline, geriatric depression, hippocampal volume, mild cognitive impairment, vascular risk, white matter hyperintensity

## Abstract

Both geriatric depression and mild cognitive impairment (MCI) confer an increased risk for the development of dementia. The mechanisms underlying the development of cognitive impairment in geriatric depression patients remain controversial. The present study aimed to explore the association of cognitive decline with vascular risk, white matter hyperintensity (WMH) burden and hippocampal volume in both remitted geriatric depression (RGD) subjects and amnestic mild cognitive impairment (aMCI) subjects. Forty-one RGD subjects, 51 aMCI subjects, and 64 healthy elderly subjects underwent multimodal MRI scans and neuropsychological tests at both baseline and a 35-month follow-up. According to the changing patterns (declining or stable) of global cognitive function during the follow-up period, each group was further divided into a declining subgroup and a stable subgroup. The Framingham 10-year cardiovascular risk, WMH volume and hippocampal volume were measured to assess vascular pathology and neurodegeneration, respectively. The RGD declining group displayed a higher vascular risk and greater WMH volume than the RGD stable group, whereas no such difference was found in the aMCI subjects. In contrast, the aMCI declining group displayed a smaller left hippocampal volume than the aMCI stable group, whereas no such difference was found in the RGD subjects. Furthermore, greater increases in the WHM volume correlated with greater decreases in global cognitive function in the RGD declining group, and greater decreases in the left hippocampal volume correlated with greater decreases in global cognitive function in the aMCI declining group. In conclusion, the cognitive decline in RGD patients is associated with vascular burden, whereas the cognitive decline in aMCI patients is associated with neurodegeneration. These findings could contribute to a better understanding of the specific mechanisms of the development of dementia in each condition.

## Introduction

Geriatric depression refers to a major depressive episode that develops in adults older than 60 years and is frequently accompanied by general impairments in physical health, global functioning, and quality of life. Geriatric depression often presents with cognitive deficits and confers an up to 50% increased risk for the development of dementia (Ownby et al., 2006; Diniz et al., 2013). Mild cognitive impairment (MCI) has been considered as a transitional state between normal aging and early Alzheimer's disease (AD), which is the most common form of dementia characterized by progressive cognitive impairment and behavioral deficits. MCI confers a high conversion rate to AD of 10–15% per year (Petersen et al., 1999). Interestingly, 11–63% of elderly MCI patients exhibit accompanying depressive symptoms, and 18–55% of depressed patients develop cognitive deficits (Panza et al., 2010). The co-existence of MCI and depression confers over twice the risk for AD as MCI alone (Modrego and Ferrandez, 2004). In view of the distinct clinical link between geriatric depression and MCI, increasing attention has been paid to the convergence and divergence of the pathogeneses of geriatric depression and MCI.

While the amyloid beta (Aβ) pathology and subsequent neurodegeneration due to tau pathology play a major role in MCI and AD pathogenesis (Albert et al., 2011), a controversy has arisen over the mechanisms underlying the cognitive impairment in geriatric depression patients. Several studies have found that the cognitive impairment in geriatric depression patients is associated with the AD-related pathology, including Aβ deposition and hippocampal atrophy (O'Brien et al., 2004; Hou et al., 2012; Byun et al., 2016). Other findings suggest that the cognitive impairment in geriatric depression patients is due to vascular pathology that is frequently represented by the white matter hyperintensity (WMH) burden in the brain (Hickie et al., 1997; Murata et al., 2001). However, these studies only explored single biomarkers in isolation and lacked an overall view of AD-related pathology and vascular pathology. Interestingly, two recent studies measured both AD-related pathology and vascular burden in geriatric depression patients with and without MCI (Diniz et al., 2015; Byun et al., 2016). However, one study found that geriatric depression subjects with MCI display greater WMH burden than geriatric depression subjects with normal cognition, suggesting that vascular pathology is related to the cognitive impairment in geriatric depression subjects (Diniz et al., 2015). The other study demonstrated that AD processes contribute to the co-existence of geriatric depression and MCI (Byun et al., 2016). Both of these studies employed cross-sectional data. A great deal of studies have revealed that cognitive impairment is improved following the remission of depressive symptoms in a significant proportion of geriatric depression patients but not in all patients (Barch et al., 2012). Thus, measuring cognitive impairment in geriatric depression patients using cross-sectional data may have increased the heterogeneity and contributed to the divergence. However, few studies have explored and compared the relationships of cognitive impairment, AD-related pathology, and vascular pathology in both geriatric depression subjects and MCI subjects despite the close link between geriatric depression and MCI. The investigation of the convergence and divergence between the two conditions could deepen the understanding of the mechanisms underlying the development of dementia in each condition.

The present 35-month longitudinal study recruited remitted geriatric depression (RGD) subjects, amnestic mild cognitive impairment (aMCI, a subtype of MCI characterized by episodic memory deficits) subjects and healthy elderly subjects. Neuropsychological tests and multimodal MRI scans were performed at both baseline and a 35-month follow-up. According to the changing patterns (declining or stable) of global cognitive function during the follow-up period, the subjects were further divided into an RGD declining group, an RGD stable group, an aMCI declining group, an aMCI stable group, a control declining group, and a control stable group. Vascular risk, WMH volume, and hippocampal volume were explored to assess vascular pathology and neurodegeneration, respectively. The present study aimed to (i) explore and compare the associations of cognitive decline with vascular risk, WMH burden and hippocampal volume in RGD subjects and aMCI subjects and (ii) determine the behavioral significance of WMH burden and hippocampal atrophy.

## Materials and methods

### Participants

As described in our previous study (Ye et al., 2017), 72 RGD patients, 87 aMCI patients, and 135 healthy elderly controls were recruited at the Affiliated ZhongDa Hospital, Southeast University. This study was performed in accordance with the recommendations of the Affiliated ZhongDa Hospital of Southeast University Research Ethics Committee with written informed consent from all subjects. All subjects provided written informed consent in accordance with the Declaration of Helsinki. The protocol was approved by the Affiliated ZhongDa Hospital of Southeast University Research Ethics Committee. The participants were followed up for 35 months on average. A total of 41 RGD subjects, 51 aMCI subjects, and 64 controls were included in the present study. All of these subjects underwent multimodal MRI scans and neuropsychological tests at both baseline and follow-up. The reasons for the loss to follow-up of the other subjects included the development of neurological or other psychiatric diseases, relocation to other cities, death, and subjective unwillingness. According to the changing patterns (declining or stable) of global cognitive function during the follow-up period, the included subjects were further divided into the following six groups: RGD declining group (*n* = 18), RGD stable group (*n* = 23), aMCI declining group (*n* = 33), aMCI stable group (*n* = 18), control declining group (*n* = 21), and control stable group (*n* = 43).

### Neuropsychological assessments

Each subject underwent a standardized diagnostic evaluation that included demographic information, medical history, and examinations of the neurological and mental statuses. Global cognitive function was assessed with the Mini Mental State Examination (MMSE), a Clinical Dementia Rating (CDR) and a Mattis Dementia Rating Scale-2 (MDRS-2). The MDRS-2 is a reliable and valid psychometric instrument for detecting and staging dementia (Vitaliano et al., 1984; Smith et al., 1994; Green et al., 1995; Monsch et al., 1995). The MDRS is also effective for tracking cognitive decline over time (Salmon et al., 1990; Galasko et al., 2000; Gould et al., 2001). Subjects with an MDRS-2 score below 130 or a loss of more than five points during the follow-up period were defined as the “declining group” (Schmidt et al., 1994; Tard et al., 2015). Other subjects were defined as the “stable group.” The mental statuses were assessed with the *Structured Clinical Interview for Diagnostic and Statistical Manual of Mental Disorders*, Fourth Edition (DSM-IV) Axis I Disorders (SCID-I), the Hamilton Depression Scale (HAMD), and the Self-Rating Depression Scale. All subjects underwent a neuropsychological battery test that included the Auditory-Verbal Learning Test-Delayed Recall (AVLT-DR), Rey-Osterrieth Complex Figure Test (CFT) with its 20-min Delayed Recall (CFT-DR), Trail Making Tests (TMT)-A and B, Stroop Color and Word Tests A, B, and C (Stroop A, B, and C), Verbal Fluency Test (VFT), Digital Span Test (DST), Semantic Similarity Test (Similarity), Digital Symbol Substitution Test (DSST), and Clock Drawing Test (CDT).

### Inclusion and exclusion criteria

The inclusion and exclusion assessments were performed by two experienced neuropsychiatric physicians who administered a structured interview to subjects and their informants. The inclusion criteria for RGD subjects were as follows: (1) the age was >60 years; (2) all subjects had previously met the DSM-IV criteria for major depression disorder and had remitted for >6 months before enrollment; (3) the duration of illness was <5 years and the period of remaining anti-depressant medication-free was >3 months before the assessment; and (4) the HAMD scores were <7 and MMSE scores were >24. The exclusion criteria were as follows: (1) primary neurological illness, including stroke or dementia; (2) another major psychiatric illness, including substance abuse or dependence; (3) history of electroconvulsive therapy; and (4) a medical illness that impaired cognitive function.

aMCI subjects were included according to the diagnostic criteria proposed by Petersen (2004) and others (Winblad et al., 2004), which was also described in our prior study (Ye et al., 2016). The inclusion criteria for the aMCI subjects were as follows: (1) subjective memory impairment corroborated by the subject and an informant; (2) objective memory performances documented by an AVLT-DR score ≤1.5 standard deviations from the age-adjusted and education-adjusted norms (the cutoff was ≤4 correct responses on 12 items for ≥8 years of education); (3) normal general cognitive function evaluated by an MMSE score ≥24; (4) a CDR of 0.5, with at least a 0.5 in the memory domain; (5) minimal or no impairment of routine daily life activities; and (6) the absence of dementia or insufficiency in meeting the National Institute of Neurological and Communicative Disorders and Stroke and the AD and Related Disorders Association (NINCDS-ADRDA) and DSM-IV criteria for AD. The exclusion criteria were as follows: (1) a history of stroke (modified Hachinski score of >4), head injury, alcoholism, epilepsy, Parkinson's disease, major depression (excluded by a self-rating depression scale), or other psychiatric or neurological illness (excluded by case history and clinical assessment); (2) major medical illness (e.g., anemia, cancer, or thyroid dysfunction); (3) severe visual or hearing loss; and (4) T2-weighted MRI showing major infarction or other lesions (two experienced radiologists executed the scans).

Control subjects were required to have a CDR of 0, an MMSE score ≥26, and a delayed recall score >4 for those with ≥8 years of education. These participants also met the aforementioned exclusion criteria for aMCI.

### Apolipoprotein E (*ApoE*) genotyping

To control for possible differences in hippocampal volume related to the *ApoE* ε4 allele (*ApoE* ε4), the most important genetic risk factor for sporadic AD, the status of *ApoE* ε4 was also assessed. Genomic DNA was extracted from 250 μL of EDTA-anticoagulated blood collected from each subject using a DNA direct kit (Tiangen, China). A polymerase chain reaction-based restriction fragment length polymorphism (PCR-RFLP) assay was employed to detect the rs7412 and rs429358 alleles, the haplotypes of which ultimately determine the *ApoE* genotype.

### Framingham 10-year cardiovascular risk

The Framingham 10-year risk for developing general cardiovascular disease (including myocardial infarction, coronary death, heart failure, angina, stroke, transient ischemic attack, and peripheral artery disease) was calculated using gender, age, systolic blood pressure, treatment for hypertension, smoking, diabetes, and body mass index (D'Agostino et al., 2008). The Framingham 10-year cardiovascular risk was assessed for each subject at baseline.

### Magnetic resonance imaging procedures

The subjects were scanned using a Siemens Verio 3.0-T scanner (Siemens, Erlangen, Germany) with a 12-channel head coil at the Affiliated ZhongDa Hospital of Southeast University. A belt and foam pads were used to immobilize their heads to minimize head motion. High-resolution T1-weighted axial images covering the whole brain were obtained by a 3D-magnetization prepared rapid gradient-echo sequence: repetition time (TR) = 1,900 ms; echo time (TE) = 2.48 ms; flip angle (FA) = 9°; acquisition matrix = 256 × 256; field of view (FOV) = 250 × 250 mm; thickness = 1.0 mm; gap = 0 mm, number of slices = 176. The T2 FLAIR axial images were obtained with the following parameters: TR = 8,000 ms; TE = 94 ms; FA = 150°; acquisition matrix = 256 × 162; thickness = 5.0 mm; gap = 0 mm, number of slices = 20.

### WMH segmentation and quantification

Lesions were segmented by the lesion growth algorithm (Schmidt et al., 2012) as implemented in the LST toolbox version 2.0.15 (www.statistical-modelling.de/lst.html) for Statistical Parametric Mapping software (SPM12, http://www.fil.ion.ucl.ac.uk/spm). The algorithm first segments the T1 images into the three main tissue classes (cerebrospinal fluid, gray matter, and white matter). This information is then combined with the coregistered T2 FLAIR intensities to calculate lesion belief maps. By thresholding these maps with a pre-chosen initial threshold (κ = 0.30) an initial binary lesion map is obtained and is subsequently grown along voxels that appear hyperintense on the T2 FLAIR image. The result is a lesion probability map. It should be noted that the κ-value was determined by the visual inspection of the results by three experienced raters.

### Hippocampal volume assessment

As described in our previous study (Bai et al., 2009), hippocampal volume analysis was performed using the VBM8 toolbox for SPM12. First, the T1 images were normalized to the Montreal Neurological Institute (MNI) template using an affine and non-linear spatial normalization and re-sampled to a voxel size of 1.5 × 1.5 × 1.5 mm. Second, the normalized images were segmented into cerebrospinal fluid, gray matter, and white matter segments according to MNI prior probability maps. Then, Jacobian modulation was applied to the segmented gray matter image, which could be incorporated to compensate for the effect of spatial normalization. Finally, the extracted gray matter set was smoothed with an 8-mm full width at half maximum Gaussian filter to reduce the effects of individual variation in gyral anatomy and to increase the signal-to-noise ratio. The hippocampus (left and right separately) was isolated using automated anatomical labeling implemented through the Resting State fMRI Data Analysis Toolkit 1.7 software (http://restfmri.net/forum/index.php). Next, the hippocampal regions were interpolated to the same dimension, sizes, and origins with individual images. Finally, a mean volume index of all of the voxels of the hippocampal region (left and right) was computed for each subject. The hippocampal volume was obtained by multiplying the mean volume index by the number of voxels and the size of each voxel (1.5 × 1.5 × 1.5 mm).

### Statistical analysis

#### Demographic and neuropsychological data

Mixed analysis of variance (ANOVA; with disease, cognitive change and time as fixed factors) and χ^2^-tests (applied for the comparisons of gender and *ApoE* ε4 status) were used to analyse the demographic data and neuropsychological data for statistically significant differences (*P* < 0.05). The individual raw scores of each cognitive test (except the MMSE and MDRS-2) were transformed into *Z* scores according to the following equation: $Z_{i}=\frac{r_{i}-m}{S}$. *Z*_*i*_ indicates the *Z* scores for the *i*th subject, *r*_*i*_ indicates the raw score for the *i*th subject, *m* indicates the average score for each test for each group, and *S* indicates the standard deviation of the test scores for each group. The neuropsychological tests were grouped into four cognitive domains, including episodic memory, executive function, visuospatial function, and information processing speed. The composite *Z* score for each cognitive domain was obtained by averaging the *Z* scores of the relevant neuropsychological tests according to the following divisions: episodic memory (two tests, including the AVLT-DR and CFT-DR), executive function (five tests, including the TMT-B, Stroop C, VFT, DST-backward, and Similarity tests), visuospatial function (two tests, including the CFT and CDT), and information processing speed (four tests, including the DSST, TMT-A, Stroop A, and Stroop B). All statistical procedures were performed with the SPSS 19.0 software (SPSS, Inc., Chicago, IL, USA).

#### Vascular risk, WMH volume, and hippocampal volume data

To improve the normal distribution of the Framingham 10-year cardiovascular risk data, a log_10_-transformation was applied to the raw data. Mixed ANOVA (with disease and cognitive change as fixed factors) was used to analyse the Framingham 10-year cardiovascular risk. Mixed analysis of covariance (ANCOVA; with disease, cognitive change, and time as fixed factors) was used to analyse the WMH volume and hippocampal volume, controlling for age, gender, and years of education. *ApoE* status was also treated as a covariate in the analysis of hippocampal volume. *Post-hoc* tests were performed to explore group differences in the ANOVA or ANCOVA. All statistical procedures utilized SPSS 19.0 software (SPSS, Inc., Chicago, IL, USA) with significance set at *P* < 0.05.

#### Correlative analysis between cognitive impairment and WMH volume or hippocampal volume

Correlation analyses were performed between the longitudinal changes of global cognitive function (MMSE and MDRS-2) and the longitudinal changes of WMH volume and hippocampal volume in RGD declining group and aMCI declining group. All statistical procedures for correlation analysis utilized SPSS 19.0 software (SPSS, Inc., Chicago, IL, USA) with significance of *P* < 0.05.

## Results

### Demographic and neuropsychological data

As shown in Table 1, no significant differences in age or years of education were noted between the six groups. There were more females in the RGD stable group. A greater percentage of *ApoE* ε4 carriers were shown in the aMCI declining group. Significant main effects of disease were found on all cognitive tests. Compared with the control subjects, both the RGD subjects and aMCI subjects displayed poorer performances in all cognitive tests with the exception of the information processing speed for the RGD subjects. Significant main effects of cognitive change were found on the MDRS-2 scores and episodic memory scores. Subjects with declining cognitive function displayed poorer performances in these two tests than the subjects with stable cognitive function. Furthermore, a main effect of time revealed longitudinal decreases in the MDRS-2 scores and executive function scores in the whole sample during the follow-up period. Finally, there was no significant interaction of disease, cognitive change and time on the neuropsychological data (data on the interactions of any two factors are not shown).

**Table 1:** Demographic and neuropsychological data.

**Items**	**Control**		**RGD**		**aMCI**		***P*****-value**			
	**Declining (*n* = 21)**	**Stable (*n* = 43)**	**Declining (*n* = 18)**	**Stable (*n* = 23)**	**Declining (*n* = 33)**	**Stable (*n* = 18)**	**Disease**	**Cognitive change**	**Time**	**Disease × cognitive change × time or χ^2^**
Age (years)	70.14 ± 6.92	69.35 ± 5.27	68.33 ± 5.97	67.13 ± 6.21	69.06 ± 7.57	67.67 ± 5.66	0.271	0.289	−	−
Education (years)	12.07 ± 3.16	13.17 ± 2.87	10.86 ± 3.03	11.35 ± 2.94	12.17 ± 3.57	11.97 ± 3.47	0.069	0.385	−	−
Gender (male: female)	10:11	19:24	8:10	4:19	23:10	10:8	−	−	−	0.008^*^
*ApoE* status (ε4 +: −)	4:17	6:37	1:17	2:21	13:20	5:13	−	−	−	0.019^*^
**MMSE**										
Baseline	28.29 ± 1.15	28.26 ± 1.45	27.56 ± 1.89	27.52 ± 1.73	26.82 ± 2.44	27.17 ± 1.69	<0.001^*^^a^^,^^b^	0.057	0.167	0.129
Follow-up	27.95 ± 1.56	28.19 ± 1.56	26.67 ± 2.28	27.30 ± 1.58	25.36 ± 3.83	28.00 ± 1.20				
**MDRS-2**										
Baseline	139.10 ± 2.26	137.44 ± 3.80	134.11 ± 5.39	136.35 ± 3.26	133.03 ± 6.56	133.28 ± 5.96	<0.001^*^^a^^,^^b^^,^^c^	< 0.001^*^	< 0.001^*^	0.074
Follow-up	131.52 ± 3.47	137.58 ± 3.73	129.28 ± 4.51	135.55 ± 2.42	124.16 ± 10.38	135.13 ± 2.64				
**EPISODIC MEMORY**										
Baseline	0.63 ± 0.44	0.46 ± 0.55	−0.33 ± 0.72	0.10 ± 0.64	−0.81 ± 0.57	−0.34 ± 0.62	<0.001^*^^a^^,^^b^^,^^c^	0.004^*^	0.913	0.718
Follow-up	0.46 ± 0.46	0.50 ± 0.65	−0.30 ± 0.70	0.07 ± 0.75	−0.77 ± 0.75	−0.32 ± 0.82				
**EXECUTIVE FUNCTION**										
Baseline	0.31 ± 0.57	0.41 ± 0.61	−0.06 ± 0.57	0.13 ± 0.47	−0.31 ± 0.56	−0.20 ± 0.64	<0.001^*^^a^^,^^b^^,^^c^	0.158	0.020^*^	0.167
Follow-up	0.14 ± 0.59	0.17 ± 0.63	−0.13 ± 0.66	−0.05 ± 0.56	−0.64 ± 0.63	−0.14 ± 0.57				
**VISUOSPATIAL FUNCTION**										
Baseline	0.37 ± 0.59	0.26 ± 0.71	−0.26 ± 0.81	−0.15 ± 0.76	−0.31 ± 0.93	−0.18 ± 0.82	0.001^*^^a^^,^^b^	0.336	0.563	0.570
Follow-up	0.27 ± 0.95	0.16 ± 0.90	−0.15 ± 0.84	−0.07 ± 1.07	−0.33 ± 1.38	0.19 ± 0.52				
**INFORMATION PROCESSING SPEED**										
Baseline	0.35 ± 0.71	0.27 ± 0.85	0.12 ± 0.75	0.32 ± 0.76	−0.46 ± 0.64	−0.24 ± 0.62	0.001^*^^b^^,^^c^	0.900	0.089	0.964
Follow-up	0.12 ± 0.64	0.12 ± 0.88	0.05 ± 0.80	0.30 ± 0.86	−0.67 ± 0.71	−0.35 ± 0.64				

*Values are presented as the means ± the standard deviations (SD). ANOVAs (with disease, cognitive change, and time as fixed factors) were applied in the comparisons of age, years of education, and neuropsychological data. χ^2^-tests were applied in the comparisons of gender and ApoE status*.

* *P < 0.05*.

a P < 0.05, RGD subjects differ from control subjects;

b P < 0.05, aMCI subjects differ from control subjects;

c *P < 0.05, RGD subjects differ from aMCI subjects*.

*Apolipoprotein E, ApoE; ANOVA, analysis of variance; MDRS-2, Mattis Dementia Rating Scale-2*.

### Framingham 10-year cardiovascular risk

**Figure 1:**
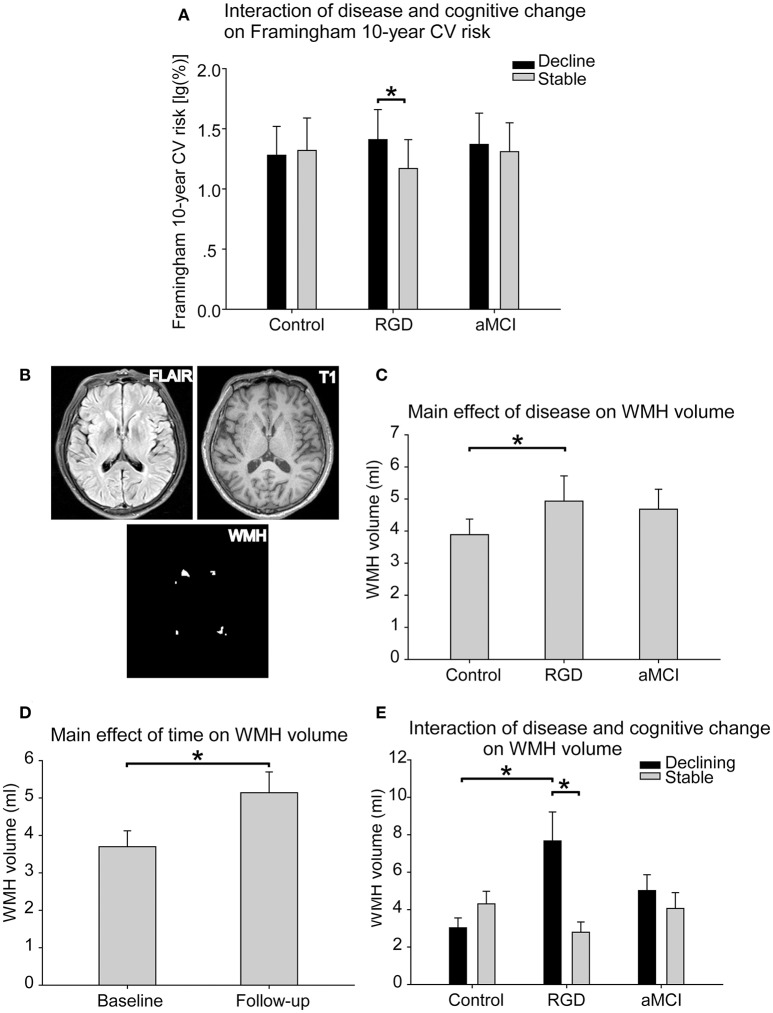
**(A)** The interaction of disease and cognitive change on the Framingham 10-year cardiovascular risk. The RGD declining group displayed a higher risk than the RGD stable group, whereas no such difference was found within the control subjects or aMCI subjects. The bars are presented with the risk values [log_10_(%)]. The error bars represent the standard errors of the means of the risk values. **(B)** The segmentation of the WMH. The WMH lesions were segmented and quantified from T2 FLAIR images and T1 images. **(C)** The main effect of disease on WMH volume. The RGD subjects had greater WMH volumes than the control subjects. **(D)** The main effect of time on WMH volume. The whole sample displayed greater WMH volume at follow-up than at baseline. **(E)** The interaction of disease and cognitive change on WMH volume. The RGD declining group displayed greater WMH volume than the RGD stable group. The RGD declining group displayed greater WMH volume than the control declining group. The bars are presented with the WMH volumes. The error bars represent the standard errors of the means of the WMH volumes. *P < 0.05. aMCI, Amnestic mild cognitive impairment; CV, cardiovascular; RGD, remitted geriatric depression; WMH, white matter hyperintensities.

As presented in Table 2, a significant interaction of disease and cognitive change was found in the Framingham 10-year cardiovascular risk. *Post-hoc* tests revealed that the RGD declining group displayed a higher risk than the RGD stable group, whereas no such difference was found within the control subjects or aMCI subjects (Figure 1A).

**Table 2:** Vascular risk, white matter hyperintensity volume, and hippocampal volume data.

**Items**	**Control**		**RGD**		**aMCI**		***P*****-value**						
	**Declining (*n* = 21)**	**Stable (*n* = 43)**	**Declining (*n* = 18)**	**Stable (*n* = 23)**	**Declining (*n* = 33)**	**Stable (*n* = 18)**	**Disease**	**Cognitive change**	**Time**	**Disease × cognitive change**	**Disease × time**	**Cognitive change × time**	**Disease × cognitive change × time**
**FRAMINGHAM 10-YEAR CV RISK [log_10_(%)]**													
Baseline	1.28 ± 0.24	1.32 ± 0.27	1.41 ± 0.25	1.17 ± 0.24	1.37 ± 0.26	1.31 ± 0.24	0.581	0.061	−	0.040^*^	−	−	−
**WHITE MATTER HYPERINTENSITY VOLUME (ml)**													
Baseline	2.62 ± 3.03	3.53 ± 5.14	6.55 ± 8.60	2.52 ± 3.50	4.19 ± 5.91	3.11 ± 3.57	0.030^*^	0.172	0.036^*^	0.003^*^	0.936	0.861	0.760
Follow-up	3.44 ± 3.75	5.09 ± 7.14	8.79 ± 9.05	3.06 ± 3.94	5.85 ± 7.73	5.01 ± 6.22							
**LEFT HIPPOCAMPAL VOLUME (ml)**													
Baseline	2.99 ± 0.27	2.95 ± 0.31	2.91 ± 0.37	2.93 ± 0.24	2.79 ± 0.45	2.99 ± 0.44	0.031^*^	0.328	0.109	0.016^*^	0.797	0.689	0.837
Follow-up	2.93 ± 0.29	2.79 ± 0.56	2.90 ± 0.44	2.89 ± 0.26	2.69 ± 0.56	2.91 ± 0.57							
**RIGHT HIPPOCAMPAL VOLUME (ml)**													
Baseline	3.15 ± 0.37	3.09 ± 0.31	3.13 ± 0.42	3.10 ± 0.24	3.00 ± 0.49	3.16 ± 0.51	0.063	0.838	0.116	0.017^*^	0.915	0.817	0.699
Follow-up	3.13 ± 0.37	2.94 ± 0.57	3.07 ± 0.48	3.06 ± 0.26	2.88 ± 0.57	3.08 ± 0.63							

*Values are presented as the means ± the standard deviations (SD). ANOVA (with disease, cognitive change, and time as fixed factors) was applied in the comparison of the Framingham 10-year CV risk. ANCOVAs (with disease, cognitive change, and time as fixed factors) were applied in the comparisons of white matter hyperintensity volume and hippocampal volume, controlling for ApoE status (only for hippocampal volume), age, gender and years of education*.

* *P < 0.05*.

*ANCOVA, Analysis of covariance; ANOVA, analysis of variance; CV, cardiovascular*.

### WMH volume

WMH lesions were segmented from T2 FLAIR images and T1 images in each subject (Figure 1B). As presented in Table 2, a significant main effect of disease on WMH volume was found. The RGD subjects exhibited greater WMH volume than the control subjects, whereas no significant difference was found between the aMCI subjects and the control subjects or between the aMCI subjects and the RGD subjects (Figure 1C). A significant main effect of time revealed that the whole sample displayed greater WMH volume at follow-up than at baseline (Figure 1D).

Notably, a significant interaction of disease and cognitive change was also demonstrated. *Post-hoc* tests as illustrated in Figure 1E, revealed the following: first, in the comparisons according to the cognitive change patterns (declining vs. stable), the RGD declining group displayed greater WMH volume than did the RGD stable group, whereas no such difference was found in the control subjects or aMCI subjects; and second, in the comparisons according to disease status (control vs. RGD vs. aMCI), the RGD declining group displayed greater WMH volume than did the control declining group.

### Hippocampal volume

#### Left hippocampal volume

**Figure 2:**
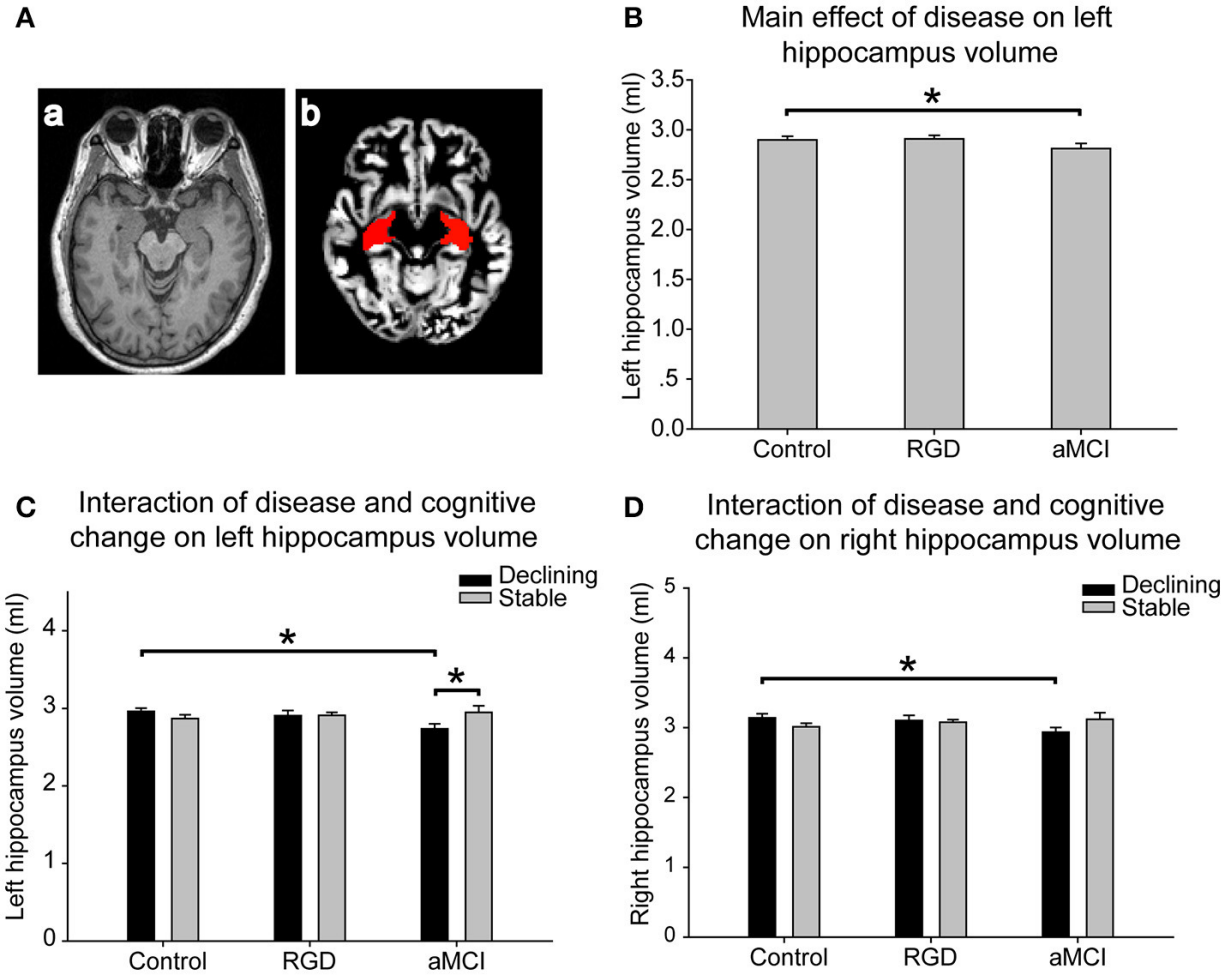
**(A)** Hippocampal volume assessment. The gray matter was segmented from T1 images **(a)**. The hippocampus, isolated using automated anatomical labeling, was interpolated to individual gray matter images **(b)**. A mean volume index of all voxels of the hippocampal regions was computed for each subject. **(B)** The main effect of disease on the left hippocampal volume. The aMCI subjects had smaller left hippocampal volumes than the control subjects. **(C)** The interaction of disease and cognitive change on the left hippocampal volume. The aMCI declining group displayed smaller hippocampal volumes than the aMCI stable group. The aMCI declining group had smaller hippocampal volumes than the control declining group. **(D)** The interaction of disease and cognitive change on the right hippocampal volume. The aMCI declining group had smaller right hippocampal volumes than the control declining group. The bars are presented with the hippocampal volumes. The error bars represent the standard errors of the means of the hippocampal volumes. *P < 0.05. aMCI, Amnestic mild cognitive impairment; RGD, remitted geriatric depression.

The mean volume index for the hippocampal region in each group was calculated by interpolating the hippocampus to the individual gray matter images segmented from the T1 images (Figure 2A). As presented in Table 2, a significant main effect of disease was found on the left hippocampal volume. The aMCI subjects had smaller left hippocampal volumes than did the control subjects, whereas no significant difference was found between the RGD subjects and the control subjects (Figure 2B). Furthermore, a significant interaction of disease and cognitive change was identified. *Post-hoc* tests revealed the following: first, in the comparisons according to cognitive change patterns (declining vs. stable), the aMCI declining group displayed smaller hippocampal volumes than did the aMCI stable group, whereas no such difference was found in the control subjects or RGD subjects; second, in the comparisons according to disease status (control vs. RGD vs. aMCI), the aMCI declining group had smaller hippocampal volumes than did the control declining group (Figure 2C).

#### Right hippocampal volume

As presented in Table 2, a significant interaction of disease and cognitive change was found in the right hippocampal volume. *Post-hoc* tests revealed the following, as illustrated in Figure 2D: first, in the comparisons according to cognitive change patterns (declining vs. stable), no significant difference was found in the control subjects, RGD subjects or aMCI subjects; second, in the comparisons according to disease status (control vs. RGD vs. aMCI), the aMCI declining group had smaller right hippocampal volumes than did the control declining group.

### Behavioral significance of WHM volume changes and hippocampal volume changes

**Figure 3:**
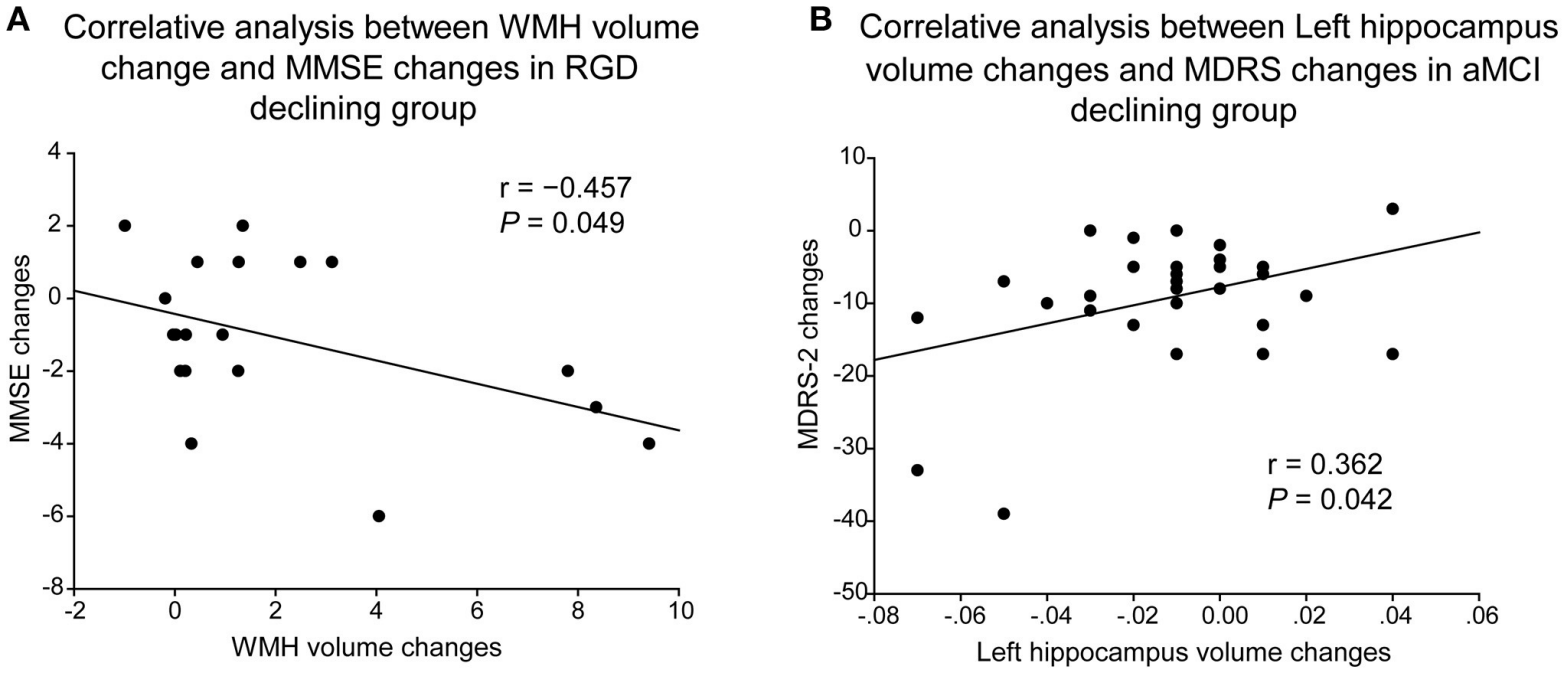
Correlative analyses of cognitive impairment with WMH volume or hippocampal volume. **(A)** Greater increases of WHM volume correlated with greater decreases of MMSE scores in the RGD declining group. **(B)** Greater decreases of the left hippocampal volume correlated with greater decreases of the MDRS-2 scores in the aMCI declining group. aMCI, Amnestic mild cognitive impairment; MDRS-2, Mattis Dementia Rating Scale-2; RGD, remitted geriatric depression.

As illustrated in Figure 3, in the RGD declining group, greater increases in WHM volume correlated with greater decreases in MMSE scores (*r* = −0.457, *P* = 0.049, two-tailed; Figure 3A). In the aMCI declining group, greater decreases in the left hippocampal volume correlated with greater decreases in the MDRS-2 scores (*r* = 0.362, *P* = 0.042, two-tailed; Figure 3B).

## Discussion

From the integrated perspective of vascular pathology and neurodegeneration, the present longitudinal study is the first to explore the association of cognitive decline with vascular risk, WMH burden and hippocampal volume in both RGD subjects and aMCI subjects. The findings revealed that the cognitive decline in RGD subjects was associated with high vascular risk and high WMH burden, whereas the cognitive decline in aMCI subjects was associated with small hippocampal volume. These findings suggest that different pathogeneses may underline the cognitive decline in RGD patients and aMCI patients.

A strength of the present study is that the 35-month longitudinal data were used to define “cognitive decline” in RGD patients and aMCI patients. As mentioned above, cognitive impairment may be improved following the remission of depressive symptoms in a portion of the population (Barch et al., 2012). Similarly, there was also a great heterogeneity in the MCI patients, although MCI has been proposed as an intermediate state between normal aging and early AD. While many MCI subjects eventually develop dementia, others remain cognitively stable, and a small proportion may actually convert to cognitively normal subjects (McKhann et al., 2011; Villemagne et al., 2011). Thus, assessing cognitive impairment using cross-sectional data may display high heterogeneity, and the definition of “cognitive decline” based on the present 35-month longitudinal data could be more reliable. RGD or aMCI subjects with cognitive decline would have a much higher risk for dementia than subjects with stable cognition.

The other strength is that the present study provided an overall view of AD-related pathology and vascular pathology in both RGD patients and aMCI patients. Studies assessing a single biomarker could show a significant association of the biomarker with cognitive impairment more easily than those that assess multiple biomarkers. As described above, previous studies that have assessed a single biomarker have demonstrated wide divergences of the association of cognitive decline with vascular burden and AD-related pathology in geriatric depression patients. A single biomarker may be associated with cognitive impairment; however, which biomarker has a major role has not been revealed by these studies. The present study explored vascular risk, WMH burden and hippocampal volume in both geriatric depression patients and aMCI patients. This overall view of AD-related pathology and vascular pathology could help to identify which pathology plays a major role in cognitive decline in each disorder.

The Framingham 10-year risk score provides an integrative perspective of multiple vascular risk factors (D'Agostino et al., 2008). In the present study, the RGD declining group had a much higher risk than did the RGD stable group. This is consentient with a previous study that demonstrated that a high score on the Framingham stroke risk scale is associated with cognitive impairment in elderly patients with major depressive disorder (Yeh et al., 2011). In contrast, the aMCI declining group and the aMCI stable group did not differ significantly in Framingham 10-year risk scores. Similarly, a 2.4-year follow-up study investigated the contribution of vascular burden to the progression of MCI to dementia and found no association between the Framingham Stroke Risk Profile scores and the progression to dementia (Clerici et al., 2012). A recent study explored the association between a vascular risk index and cognitive outcomes among MCI subjects and cognitively normal elderly subjects using linear regression models. Increased Framingham Stroke Risk Profile scores for MCI subjects only correlated with worse longitudinal episodic memory and not with the global cognition or other cognitive domains (Jefferson et al., 2015). Together with these findings, the present study highlighted the association of cognitive decline with vascular risk in geriatric depression by investigating the link in both RGD subjects and aMCI subjects.

The burden of small vessel cerebrovascular disease is well represented by WMH lesions on T2 FLAIR images. In the present study, greater WMH volume was found in the RGD subjects than in the control subjects. These findings support the vascular depression hypothesis in which cerebrovascular disease may predispose, precipitate, or perpetuate geriatric depression; this hypothesis is also known as “vascular depression” (Alexopoulos et al., 1997). In addition to geriatric depression, the cognitive decline in the RGD subjects was also related to WMH burden as suggested by the difference between the RGD declining group and the RGD stable group. The RGD declining group displayed greater WMH volume than did the RGD stable group, and greater increases in the WHM volume correlated with greater decreases of global function in the RGD declining group. This is consistent with previous findings that greater WMH burden is associated with cognitive deficits in geriatric depression patients (Hickie et al., 1997; Murata et al., 2001; Diniz et al., 2015). A significant advance of the present study is that the vascular burden was represented by both vascular risk and WMH volume. The present consistent findings in vascular risk and WMH volume convincingly relate the vascular burden to cognitive decline in geriatric depression patients. Indeed, increasing attention has been given to the concept of “disconnection syndromes” in which extensive ischemia and white matter lesions result in depression and cognitive deficits by disrupting cortical-subcortical or cortical-cortical connections (Alexopoulos, 2002).

However, the present study did not find a significant difference in WMH volume between the aMCI declining group and the aMCI stable group. A previous study also demonstrated that WMH is not associated with cognition in MCI subjects (Chen et al., 2006). However, other studies have demonstrated that greater WMH is associated with poor episodic memory and slow processing speed in MCI subjects (Fujishima et al., 2014; Lorius et al., 2015). Greater WMH is related to rapid cognitive decline in MCI subjects (Tosto et al., 2014). Furthermore, severe WMH also predicts the progression from MCI to dementia (Prasad et al., 2011; Kim et al., 2015). The divergences may be due to two reasons. First, the present follow-up period may not have been long enough to generate significant differences in WMH volume between the aMCI declining group and the aMCI stable group, although the aMCI declining group had moderately, but not significantly, greater WMH volumes than did the aMCI stable group. We will continue to follow up these subjects. Second, the analysis of WMH volume was performed in RGD subjects, aMCI subjects, and control subjects using an ANCOVA, with which it was more difficult to show differences than with the analyses used for the aMCI subjects alone or in the comparison between the aMCI subjects and the control subjects. Nevertheless, the present findings highlight a major role of vascular burden in the mechanisms underlying cognitive decline in RGD patients and suggest a secondary role of vascular burden in cognitive decline in aMCI patients.

Atrophy of the hippocampus, which is one of the earliest brain areas developing AD-related pathology, indicates the degree of neurodegeneration in MCI patients and serves as a well-established indicator for the early diagnosis of AD (Aisen et al., 2010; Jack et al., 2010). The present study revealed that the aMCI subjects had smaller hippocampal volumes than the controls, and the aMCI declining group had smaller hippocampal volumes than the aMCI stable group and/or control group. Furthermore, greater decreases in the left hippocampal volume correlated with greater decreases of global cognitive function in the aMCI declining group. In contrast, no significant difference in hippocampal volume was found between the RGD declining group and the RGD stable group. A handful of studies have explored the relationship between hippocampal volume and cognitive impairment in geriatric depression patients. Smaller hippocampal volumes are associated with impairments in global cognitive function (Sachs-Erisson et al., 2011), memory (O'Brien et al., 2004), and executive function (Hou et al., 2012) in geriatric depression patients. A recent study demonstrated that hippocampal volume is not associated with executive dysfunction, i.e., the common cognitive deficit in geriatric depression, although both smaller hippocampal volume and poor cognitive performance are displayed in depressed patients. The study suggested that the link between hippocampal volume and executive dysfunction may be indirect (Khan et al., 2015). The present data did not support the association between hippocampal volume and global cognitive function in RGD patients. These divergences may come from the heterogeneities of geriatric depression populations and methodologies, including statistical methods and the assessment and definition of cognitive impairment/decline.

Several limitations should be addressed. First, the present study did not measure Aβ deposition, which is a major hallmark of the pathological changes in AD. Increasing evidence suggests that vascular pathology interacts with Aβ pathology. Cerebral ischaemia promotes the accumulation of Aβ, and Aβ deposition in turn results in further decreases in cerebral blood flow (Popa-Wagner et al., 2015). The coexistence of vascular pathology and Aβ pathology is very common in dementia (Saito and Murayama, 2007; Schneider et al., 2009). Thus, although the present study demonstrated that different pathologies were associated with the cognitive decline in RGD patients and aMCI patients, there may also be convergent pathologies contributing to the cognitive decline in both conditions. Indeed, our previous study found both convergent and divergent patterns of microstructural integrity of white matter between RGD and aMCI subjects (Bai et al., 2012). Second, the range of the declines in the MDRS-2 scores in the RGD declining group, aMCI declining group and control declining group were not matched. Thus, the results of comparisons of WMH volume and hippocampal volume between these three groups should be treated with caution. Finally, the present study did not measure the Aβ pathology and tau pathology using PET imaging or cerebrospinal fluid analyses of Aβ and Tau. Some control subjects, especially those with cognitive decline, might also have AD-related pathology and could not serve as a control group. Some control declining subjects may improve their performance later on. Similarity, not all of aMCI subjects with cognitive decline would display AD-related pathology and ultimately develop dementia.

In conclusion, the present findings suggest that cognitive decline in RGD patients is associated with vascular burden, whereas the cognitive decline in aMCI patients is associated with neurodegeneration. These findings could contribute to a better understanding of specific mechanisms involved in the development of dementia in each condition, which may further provide novel strategies for the prevention and treatment of dementia.

## Ethics statement

This study was carried out in accordance with the recommendations of the Affiliated ZhongDa Hospital of Southeast University Research Ethics Committee with written informed consent from all subjects. All subjects gave written informed consent in accordance with the Declaration of Helsinki. The protocol was approved by the Affiliated ZhongDa Hospital of Southeast University Research Ethics Committee.

## Author contributions

FB: Study design, interpretation, data analysis, and manuscript writing. ZZ, HZ, and CX: Study design and interpretation. QY: Study design, data collection, analysis, and manuscript writing. FS, LG, HS, and WL: Data collection and analysis.

### Conflict of interest statement

The authors declare that the research was conducted in the absence of any commercial or financial relationships that could be construed as a potential conflict of interest.
